# Akute Polyarthritis nach Karibikaufenthalt

**DOI:** 10.1007/s00108-024-01773-5

**Published:** 2024-09-17

**Authors:** Johannes Mayer, Michaela Köhm, Matthias Wahle

**Affiliations:** 1 3. Med. Klinik, Sektion Rheumatologie & Klinische Immunologie, Universitätsklinikum Augsburg, Stenglinstraße 2, 86156 Augsburg, Deutschland; 2 Medizinische Klinik II, Abteilung Rheumatologie, Universitätsklinikum Frankfurt, Goethe-Universität, Theodor-Stern-Kai 7, 60596 Frankfurt am Main, Deutschland

**Keywords:** Arthritis, Reiseinfektion, Chikungunya-Virus, Nicht‐Steroidale Antirheumatika, Methotrexat, Arthritis, Travel‐related infection, Chikungunya‐Virus, Non‐steroidal anti‐rheumatic drugs, Methotrexate

## Abstract

Eine 59-jährige Patientin wird wegen einer akut aufgetretenen Polyarthritis nach Karibikreise vorgestellt. Begleitend bestehen konstitutionelle Symptome sowie Myalgien und Arthralgien. In der Bildgebung zeigt sich eine Synovitis der Hand- und Fingergrundgelenke ohne erosive Veränderungen. Immunserologisch stellen sich zudem normwertige Befunde ohne Hinweis auf Autoimmunerkrankung oder Vaskulitis dar. In der erweiterten Diagnostik zeigen sich serologische Hinweise für eine Infektion mit dem Chikungunya-Virus.

## Abstract (en)

A 59-year-old female patient presented with acute polyarthritis after a holiday in the Caribbean. In addition, constitutional symptoms as well as myalgia and arthralgia were reported. Imaging demonstrated synovitis of the wrist and fingers without erosive changes. Immunoserological findings were normal with no evidence of autoimmune disease or vasculitis. Further evaluation revealed serological evidence of chikungunya virus infection.

## Kasuistik

### Anamnese

Die Vorstellung der 59-jährigen Patientin erfolgte wegen akut auftretender, an Händen und Füßen betonter Gelenkschmerzen. Diese nahmen im Verlauf einen immobilisierenden Charakter an.

Vorausgegangen war eine Urlaubsreise in die Dominikanische Republik. Im Verlauf des Aufenthalts trat bei der Patientin akutes Fieber bis ca. 39 °C mit darauffolgenden Arthralgien und Myalgien auf. Vor Ort erfolgte eine symptomatische Therapie der Beschwerden. Nach Reiserückkehr nahmen die Arthralgien in den Händen und Füßen zu. Aufgrund der Beschwerden erfolgte eine Vorstellung in einem auswärtigen Krankenhaus. Dort wurde eine umfangreiche rheumatologische und infektiologische Serumdiagnostik (u. a. Denguevirus, CMV, EBV, Hepatitisviren) ohne konkreten Hinweis für eine Ursache durchgeführt.

Im Röntgen von Thorax sowie Händen und Füßen wurden regelrechte Befunde erhoben, eine Abdomensonographie war unauffällig.

Die Patientin wurde symptomatisch mit nichtsteroidalen Antirheumatika (NSAR) behandelt und im Verlauf entlassen. Aufgrund der weiter progredienten Symptomatik stellte sie sich akut fachrheumatologisch vor. Vorerkrankungen oder eine regelmäßige Medikation bestanden nicht.

### Klinischer Befund

**Abb. 1:**
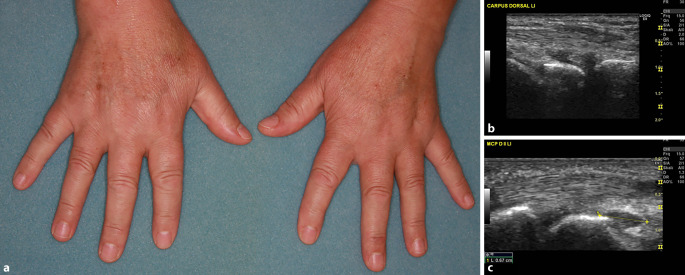
Klinischer Befund mit Schwellung der Hand‑, Karpal- und MCP-Gelenke beider Hände (**a**). Synovitis des Carpus und des MCP-2-Gelenks links in der Arthrosonographie (**b**, **c**)

In der klinischen Untersuchung zeigten sich eine Gangstörung, generalisierte Muskelschmerzen und eine deutliche Schmerzhaftigkeit der Hände und Füße. Schwellungen und Druckschmerzen waren vor allem über den Metakarpophalangeal(MCP)- und Handgelenken (Abb. 1a), den Metatarsophalangeal(MTP)-Gelenken und den oberen Sprunggelenken nachweisbar. Es bestand ein proximaler muskulärer Druckschmerz an den Armen. Fieber oder Hautveränderungen lagen nicht vor. Der weitere klinische Untersuchungsbefund war regelrecht.

### Arthrosonographie

In der Untersuchung der Hände zeigte sich eine Synovitis II° in den Handgelenken sowie in den MCP-2- und -3-Gelenken beidseits (Abb. 1b, c). Eine Power-Doppler-Aktivität bestand nicht, Erosionen waren nicht nachweisbar, degenerative Gelenkveränderungen gering ausgeprägt. An den Beugesehnen der Hände mäßig floride Tenosynovitis.

### Laborbefunde

Laborchemisch war das C‑reaktive Protein mit 0,81 mg/dl (Normwert < 0,5) und die BSG mit 28 mm/h (NW < 20) leicht erhöht, antinukleäre Antikörper (ANA), antineutrophile zytoplasmatische Antikörper (ANCA), Rheumafaktor und Anti-citrulliniertes-Protein-Antikörper (ACPA) zeigten Normalbefunde.

In der ergänzenden infektionsserologischen Diagnostik waren die Parvovirus-B19- und HIV-Serologie unauffällig, demgegenüber waren IgM-Ak gegen das Chikungunya-Virus (CHIKV) positiv und IgG-Ak hochtitrig nachweisbar.

### Diagnose

-  Akute Polyarthritis bei Chikungunya-Virus-Infektion

### Therapie und Verlauf

Bei der Patientin erwies sich eine Therapie mit NSAR als nicht ausreichend, sodass die Behandlung um oral verabreichte Glukokortikoide sowie einen Protonenpumpeninhibitor erweitert wurde. Da eine Besserung der Beschwerden nur langsam eintrat, wurde eine steroidsparende Therapie mit Methotrexat 15 mg/Woche subkutan eingeleitet. Im Verlauf konnte Prednisolon ausgeschlichen werden, auch Methotrexat wurde bei zunehmender Besserung der Beschwerden unterbrochen.

### Diskussion

Die Ursachen akut bzw. subakut auftretender Polyarthritiden betreffen neben entzündlich-rheumatischen Erkrankungen auch polyartikuläre Manifestationen stoffwechselbedingter Arthritiden und Infektionen mit (reaktiver) Polyarthritis.

Die Frequenz reiseassoziierter Arbovirosen hat in den letzten 20 Jahren signifikant zugenommen [4]. Chikungunya leitet sich von einer Redewendung der Makonde (Volk im Südosten Tansanias) ab und bedeutet übersetzt „das, was sich beugt“. Akut einsetzendes Fieber, Exanthem, Konjunktivitis, Myalgien und Arthritiden prägen das klinische Spektrum von CHIKV-Infektionen [6]. CHIKV ist ein Alphavirus mit einsträngiger positiver RNA aus der Familie der Togaviren. Die westafrikanische, die ost-, zentral- und südafrikanische sowie die asiatische Viruslinie unterscheiden sich genotypisch voneinander, ihre Namensgebung bezieht sich auf deren ursprüngliche geografische Begrenzung [10]. Der Erreger wurde erstmals nach einem Ausbruch 1952–1953 in Tanganyika (Tansania) beschrieben und später als Virus identifiziert [9]. CHIKV zirkuliert in zwei Übertragungszyklen:

1. Durch *Aedes*-Mücken im enzootischen Kreislauf sylvatischer Regionen der Subsahara zwischen nichthumanen Primaten und anderen Wirbeltieren
2. Im urbanen Kreislauf durch die *Aedes*-Spezies *A. aegypti* und *A. albopictus*, die hoch anthropophil sind [2]

CHIKV-Infektionen treten in Afrika, Asien, der Karibik, sowie Zentral- und Südamerika auf [3]. In Europa konnten lokale Ausbrüche von CHIKV-Infektionen in Italien (2007 und 2017) nachgewiesen werden [8]. Nach einer Inkubationszeit von drei bis sieben Tagen (Spanne von zwei bis zwölf Tagen) kann der Verlauf in drei klinische Stadien aufgeteilt werden:

1. Die akut febrile, virämische Phase (bis zum 21. Tag)
2. Die postakute Phase bis zum Ende des dritten Monats
3. Die chronische Phase danach [12]

Da eine kausale antivirale Therapie bei CHIKV-Infektionen aktuell nicht existiert, orientiert sich die Behandlung an den klinischen Stadien:

In der Akutphase symptomatische Therapie (Analgetika nach WHO-Stufenschema). Glukokortikoide werden in der akuten Phase der Infektion nur nach kritischer Nutzen-Risiko-Abwägung empfohlen [5]. Eine Verbesserung der Gehfähigkeit und der Gelenk- und Weichteilschwellung im Vergleich zur Kontrollgruppe *(NSAR-Monotherapie)* konnte mit Ribavirin gezeigt werden [7].

In der postakuten Phase werden Glukokortikoide bei florider Arthritis eingesetzt (initial 10–20 mg Prednisolonäquivalent/Tag oral). Dosis und Therapiedauer sollten so niedrig wie möglich gewählt werden. In refraktären Fällen wird eine Therapie mit Methotrexat (MTX, 10–25 mg pro Woche) empfohlen [5]. Alternativ kann Sulfasalazin, ggf. in Kombination mit MTX, verabreicht werden. Eine große Zahl der Betroffenen (ca. 14–33 %) litt teils noch Monate nach der Infektion an einer Arthritis. Daher sollte eine strukturierte und zielorientierte Therapie nicht zu spät im Krankheitsverlauf indiziert werden (z. B. nach der akuten Phase und persistierenden Beschwerden).

Inzwischen steht auch eine Impfung gegen CHIKV zur Verfügung (die Zulassung in den USA ist erfolgt, für den europäischen Raum wird diese von der EMA empfohlen). Weitere Impfstoffkandidaten befinden sich in der Entwicklung. Daher ist mit einer Verbesserung der Prophylaxe von CHIKV-Infektionen, insbesondere bei Aufenthalt in Risikogebieten, zu rechnen [1, 11].

## Fazit für die Praxis

- Bei akut auftretenden Arthritiden sollten auch infektiöse Ursachen in Betracht gezogen werden. Infektionsassoziierte Polyarthritiden sind in erster Linie viral bedingt.
- Eine detaillierte Anamnese mit möglichen Expositionsszenarien (z. B. vorangegangene Transfusionen von Blutprodukten oder Fernreisen) erleichtert die Einordnung entsprechender Konstellationen wesentlich.
- Mit einer zunehmenden Häufigkeit von ursprünglich in wärmeren Regionen vorkommenden Virusinfektionen im europäischen und insbesondere mitteleuropäischen Raum ist durch klimatische Veränderungen zu rechnen.
